# CircIDH2 Modulates Porcine Adipogenesis via the miR-193a-5p/*RASGRP*4 Axis: Implications for ceRNA-Mediated Regulation of Fat Deposition

**DOI:** 10.3390/cells14161265

**Published:** 2025-08-15

**Authors:** Meng Li, Jiayi Chen, Wu Bao, Shuangji Ma, Mingxin Wen, Yuqi Han, Wanfeng Zhang, Yang Yang, Xiaohong Guo, Bugao Li

**Affiliations:** College of Animal Science, Shanxi Agricultural University, Jinzhong 030801, China; 13994576150@163.com (M.L.); 15735347824@163.com (J.C.); 18437985978@163.com (W.B.); 19834541517@163.com (S.M.); 15053668507@163.com (M.W.); 18335490986@163.com (Y.H.); b20201040@stu.sxau.edu.cn (W.Z.); yangyangyh@163.com (Y.Y.); g_xiaohong@126.com (X.G.)

**Keywords:** circIDH2, miR-193a-5p, *RASGRP*4, adipogenesis, ceRNA

## Abstract

Adipose tissue development plays a critical role in determining carcass quality and meat production efficiency in swine; however, the regulatory mechanisms governing fat deposition remain incompletely understood. Circular RNAs (circRNAs), characterized by high stability and resistance to RNase R degradation, have emerged as important epigenetic regulators of livestock traits. This study investigated the regulatory role of circIDH2 in adipogenic differentiation of porcine preadipocytes and the underlying molecular mechanisms. Functional assays revealed that silencing circIDH2 markedly promoted preadipocyte proliferation while inhibiting differentiation and lipid accumulation; conversely, circIDH2 overexpression produced the opposite effects. Mechanistically, circIDH2 acted as a molecular sponge for miR-193a-5p through complementary base pairing, thereby relieving the repression of its target gene *RASGRP*4, a positive regulator of adipogenesis. Furthermore, this study demonstrated that miR-193a-5p promoted proliferation but suppressed the differentiation of porcine preadipocytes, whereas *RASGRP*4 inhibited proliferation while promoting adipogenic differentiation. Rescue experiments further confirmed the regulatory relationship among circIDH2, miR-193a-5p, and *RASGRP*4. In summary, the findings indicated that circIDH2 functioned as a key regulator of adipogenesis by modulating the miR-193a-5p/*RASGRP*4 axis, thereby suppressing preadipocyte proliferation and promoting adipogenic differentiation. These results provide a theoretical foundation for future investigations into the regulatory mechanisms of adipose tissue development.

## 1. Introduction

As the world’s largest producer and consumer of pork, China’s swine industry plays a central role in the nation’s livestock production system. Enhancing meat yield and improving pork quality have become critical priorities for both producers and consumers. Circular RNAs (circRNAs), defined by their covalently closed structure that confers high stability and resistance to RNase R degradation [1], have emerged as key epigenetic regulators of economically important traits in livestock [2]. Therefore, elucidating the molecular mechanisms by which circRNAs regulate porcine adipose tissue development is of great significance for improving pork production efficiency and promoting sustainable livestock husbandry.

Emerging evidence has demonstrated that circRNAs play a pivotal role in the regulation of lipid metabolism. For example, circRNA_0046367 competitively binds to miR-34a, alleviating its transcriptional repression of PPARα, thereby significantly enhancing fatty acid β-oxidation and reducing hepatocellular lipid accumulation [3]. Similarly, circRRM2 inhibits triglyceride synthesis by modulating the miR-142-5p/NRG1 signaling axis, while circLDLR facilitates cholesterol reverse transport through the miR-667-5p/SIRT1 pathway [4,5]. Notably, circRNA_0001805 exhibits multitarget regulatory capacity by simultaneously modulating miR-106a-5p and miR-320a, leading to the inhibition of ABCA1-mediated lipogenesis and activation of CPT1-dependent β-oxidation [6]. These findings highlight the essential role of circRNAs as key regulators of lipid homeostasis. Nevertheless, a significant knowledge gap persists regarding the regulatory mechanisms of circRNAs in porcine adipose deposition and their relationship with economically important traits in agriculture.

In a previous whole-transcriptome sequencing analysis of skeletal muscle across different developmental stages in fatty-type Mashen pigs and lean-type large white pigs, a set of differentially expressed circRNAs was identified. Among them, circIDH2 was characterized as a circular RNA generated through back-splicing of exons 2–4 of the isocitrate dehydrogenase 2 (IDH2) gene [7]. IDH2 encodes a mitochondrial enzyme that plays a key role in the tricarboxylic acid cycle by catalyzing the oxidative decarboxylation of isocitrate to produce α-ketoglutarate and NADPH [8]. Previous research has demonstrated that IDH2 regulates mitochondrial oxidative phosphorylation, glutamine metabolism, and adipogenesis by influencing the levels of α-ketoglutarate and NADPH [9]. Notably, Cao et al. demonstrated that IDH2 was essential for brown adipose tissue formation, with its deficiency significantly impairing adipocyte differentiation and lipid accumulation [10]. According to the known functional roles of the host gene IDH2 and predictive analysis of circIDH2-associated regulatory networks, we hypothesize that circIDH2 epigenetically regulates fat deposition in pigs. To test this hypothesis, the present study explores the functional roles and underlying regulatory mechanisms of circIDH2 in porcine preadipocytes to provide mechanistic insights into circRNA-mediated fat deposition in swine.

## 2. Materials and Methods

### 2.1. Cell Isolation, Culture, and Transfection

Porcine preadipocytes were isolated from subcutaneous adipose tissue of neonatal piglets (Duroc × Landrace × large white pigs; sourced from Jinmin Animal Husbandry Technology Co., Ltd., Taigu, Jinzhong, China; n = 10) using collagenase digestion with collagenase type II (Thermo Fisher Scientific, Waltham, MA, USA), cells from these individual pigs were pooled together to create a single homogeneous cell population. All porcine preadipocytes used in this study were primary cells isolated directly from subcutaneous adipose tissue by our research group. The cells were cultured in DMEM/F-12 medium (Gibco™, Waltham, MA, USA) supplemented with 10% fetal bovine serum (Gibco™, Waltham, MA, USA) and 1% penicillin-streptomycin (Solarbio Life Sciences, Beijing, China) under standard conditions (37 °C, 5% CO_2_). Adipogenic differentiation was induced by treating cells with an induction medium containing 0.5 mM 3-isobutyl-1-methylxanthine (Sigma, St. Louis, MO, USA), 1 μM dexamethasone (Sigma), 10 μg/mL insulin (Sigma), and 100 μM indomethacin (Sigma, St. Louis, MO, USA) for 48 h. This process was followed by a maintenance phase in a medium containing 10 μg/mL insulin for 6 days, with medium changes every 48 h. Plasmid transfections used Lipofectamine 3000 (Gibco™, Waltham, MA, USA) with 2.5 μg DNA per 35 mm well; siRNA transfections employed RNAFit (HanBio, Shanghai, China) with 50 nM siRNA. Regarding the *RASGRP*4 gene, we designed three interference sequences (HanBio, Shanghai, China). The sequences are as follows: si-1 (sense: 5′-GGAAAGACAGCAAGAGGAAGUTT-3′, antisense: 5′-UUCCUCUUGCUGUCUUUCCUGTT-3′); si-2 (sense: 5′-GUUAGAAGAGGUUAUAGGUCG-3′, antisense: 5′-ACCUAUAACCUCUUCUAACUG-3′); si-3 (sense: 5′-GGCAAAAAGUGCAAAGUGUCC-3′, antisense: 5′-ACACUUUGCACUUUUUGCCCA-3′). The si-NC represents negative control of siRNA. The interfering sequence targeting circIDH2 (sh-circIDH2: 5′-ACAACACAGATGAGCTCAT-3′) and its negative control (sh-NC) were designed by GenePharma (Shanghai, China). For circIDH2 overexpression, the construct (OE-circIDH2) and its negative control (OE-NC) were designed by HanBio (Shanghai, China). Cells were transfected at approximately 60% confluence, and transfection efficiency was assessed 48 h post transfection using quantitative real-time polymerase chain reaction (qRT-PCR).

### 2.2. RNA Extraction and qRT-PCR

Total RNA was extracted from cells using TRIzol^®^ Reagent (Life Technologies, Carlsbad, CA, USA), followed by chloroform–isopropanol phase separation and two washes with 75% ethanol to remove impurities. RNA purity was assessed using a NanoDrop 2000 (Thermo Fisher Scientific, Waltham, MA, USA) spectrophotometer. For circRNA analysis, linear RNAs were digested with RNase R (Epicentre, Madison, WI, USA) to enhance detection specificity. cDNA synthesis was conducted using the PrimeScript^®^ RT Kit (TransGen Biotech, Beijing, China) for mRNA and circRNA, and a poly(A)-tailing-based miRNA-specific cDNA synthesis kit (Vazyme, Nanjing, China) for miRNA. QRT-PCR was performed using SYBR Premix Ex Taq II (Takara, Kusatsu, Japan) on a Bio-Rad CFX Connect system, with three technical replicates per sample. QRT-PCR was performed in a 10 μL reaction volume containing 5 μL of 2× Green qPCR SuperMix, 0.5 μL each of forward and reverse primers, and 4 μL of cDNA. The amplification conditions were as follows: initial denaturation at 94 °C for 5 min; followed by 40 cycles containing denaturation at 94 °C for 15 s, annealing at 55–65 °C for 15 s, and extension at 72 °C for 20 s; followed by a final extension at 72 °C for 5 min. All primers were designed using NCBI Primer-BLAST and synthesized by Sangon Biotech (Shanghai, China). *18S rRNA* was used as the endogenous control for mRNA and circRNA, while *U6* served as the control for miRNA. Primer sequences are listed in Table S1. Relative expression levels were calculated using the 2^−ΔΔCt^ method.

### 2.3. Cell Proliferation Analysis

Cell proliferative capacity was assessed using the CCK-8 assay kit (Solarbio Life Sciences, Beijing, China). Cells were seeded into 96-well plates, and 24 h post transfection, 10 μL of CCK-8 reagent was added to each well. After a 3 h incubation period, optical density at 450 nm was measured using a microplate reader (SpectraMax iD5, Molecular Devices, San Jose, CA, USA). To further evaluate cell proliferation, the kFluor555 Click-iT EdU Imaging Kit (Solarbio Life Sciences, Beijing, China) was employed. Cells were seeded into 24-well plates and allowed to reach 70–80% confluence following transfection. EdU incorporation and detection were performed according to the manufacturer’s instructions, and fluorescent images were captured using a fluorescence microscope (Life Technologie, Carlsbad, CA, USA). Finally, the percentage of EdU-positive cells (those exhibiting specific fluorescent signal within the nucleus) relative to the total number of DAPI-stained nuclei was quantified by automated image analysis software using five representative fields of view per sample, which covered >80% of the central well area while excluding peripheral regions prone to edge effects.

### 2.4. Western Blot

Cell samples were lysed on ice for 30 min using RIPA buffer (Beyotime, Shanghai, China) containing protease inhibitors, then centrifuged at 12,000× *g* for 10 min to collect supernatants. Proteins were denatured by boiling with loading buffer (Sangon Biotech, Shanghai, China) at 100 °C for 10 min. Equal amounts of protein were separated via SDS-PAGE (120 V for 50 min, until bromophenol blue reached the gel bottom). Target protein bands (PPARγ, 57 kDa; β-actin, 42 kDa) were identified using pre-stained protein markers (PageRuler™ Prestained Protein Ladder, Thermo Fisher Scientific) and transferred onto nitrocellulose membranes (Millipore, Burlington, MA, USA) using a Bio-Rad transfer system (90 V for 90 min, ice-cooled). Membranes were blocked with 5% non-fat milk (BD Biosciences, San Jose, CA, USA) for 1 h at room temperature, then incubated with PPARγ antibody (dilution ratio 1:1000, Cat# ab310323, Abcam, Cambridge, UK) and β-actin (dilution ratio 1:1000, Cat# ab6276, Abcam, Cambridge, UK) overnight at 4 °C. After washing, membranes were incubated with secondary antibody (dilution ratio 1:10,000, Cat# ab97051, Abcam, Cambridge, UK) for 1 h at room temperature in the dark. Protein–antibody complexes were detected using the enhanced chemiluminescence substrate SuperSignal™ West Femto (Thermo Fisher, Cat# 34095, Waltham, MA, USA) and imaged with a Tanon chemiluminescence imaging system. Exposure times were adjusted between 10 s and 1 min based on signal intensity. Band intensities were quantified using ImageJ (Version 1.54p) software.

### 2.5. Oil Red O Staining for Lipid Droplet Detection

Cells were seeded in 6-well plates and induced to undergo adipogenic differentiation after reaching confluence. Once lipid droplets became prominent, the cells were washed with PBS and fixed with 4% paraformaldehyde (Sigma, St. Louis, MO, USA) for 20 min, followed by rinsing with PBS. Graded dehydration was performed through the addition of 1 mL of 60% isopropanol (Sigma, St. Louis, MO, USA) per well for 20 s, after which the solution was discarded. The cells were then stained with a filtered (0.45 μm syringe filter) Oil Red O working solution (Sigma, St. Louis, MO, USA) for 20 min under light-protected conditions. Following staining, the solution was removed, and the cells were rinsed three times with PBS, briefly treated with 60% isopropanol, and finally rinsed with PBS. Images were captured using an optical microscope (Life Technologies, Carlsbad, CA, USA). Lipid deposition was evaluated by statistically analyzing the relative expression level of Oil Red O staining (relative to the control group), specifically by measuring the total area of red (Oil Red O-positive) regions in the field of view. Statistical results were obtained using five representative fields of view per sample, covering >80% of the central well area while excluding peripheral regions prone to edge effects.

### 2.6. Dual-Luciferase Reporter Assay

To validate the interaction between circIDH2/*RASGRP*4 and miR-193a-5p, wild-type (psi-circIDH2/*RASGRP*4-WT) and mutant (psi-circIDH2/*RASGRP*4-MUT) reporter vectors were constructed based on RNAhybrid-predicted binding sites. HEK293T cells (Vazyme, Nanjing, China) at 70% confluence were co-transfected in 24-well plates with 200 ng of either WT or MUT vector and 50 nM miR-193a-5p mimics (HanBio, Shanghai, China) or negative control mimics (HanBio, Shanghai, China) using Lipofectamine 3000 (Gibco™, Waltham, MA, USA). After 48 h, cells were lysed with Passive Lysis Buffer (HanBio, Shanghai, China), and lysates were transferred to a 96-well plate. Firefly luciferase activity was measured through the addition of Luciferase Assay Reagent II (HanBio, Shanghai, China) followed by a 10 min incubation in the dark; then, Renilla luciferase activity was detected using Stop & Glo Reagentv (HanBio, Shanghai, China) with an additional 10-min incubation. The ratio of Firefly to Renilla luminescence was normalized to the WT + mimics NC group. Experiments were performed with three biological replicates, and statistical significance was determined through one-way analysis of variance (ANOVA).

### 2.7. Statistical Analysis

Data are presented as mean ± SE. Statistical significance was evaluated using Student’s *t*-test or one-way ANOVA followed by Duncan’s post hoc test in SPSS 22.0. Graphs were generated with GraphPad Prism 8.0. Statistical significance is indicated as * *p* < 0.05 and ** *p* < 0.01.

## 3. Results

### 3.1. CircIDH2 Suppresses Porcine Preadipocyte Proliferation and Promotes Adipogenic Differentiation

**Figure 1:**
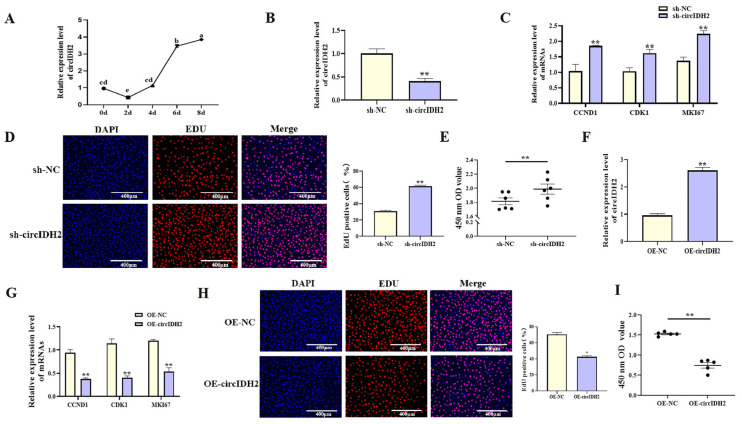
circIDH2-mediated regulation of proliferation in porcine preadipocytes. (**A**) The expression patterns of circIDH2 during adipogenic differentiation in porcine preadipocytes (n = 3). (**B**) Efficiency of circIDH2 interference (n = 3). (**C**) Expression of proliferation-related genes after interference (n = 3). (**D**) EdU staining after interference (n = 3). (**E**) CCK-8 assay results following interference (n = 6). (**F**) Efficiency of circIDH2 overexpression (n = 3). (**G**) Expression of proliferation-related genes after overexpression (n = 3). (**H**) EdU staining after overexpression (n = 3). (**I**) CCK-8 assay results following overexpression (n = 6). Abbreviations: sh-circIDH2, short hairpin RNA targeting circIDH2; sh-NC, negative control of short hairpin RNA; OE-circIDH2, overexpressing circIDH2; OE-NC, overexpressing negative control. Different lowercase superscript letters indicate significant differences (*p* < 0.05). * *p* < 0.05 and ** *p* < 0.01.

**Figure 2:**
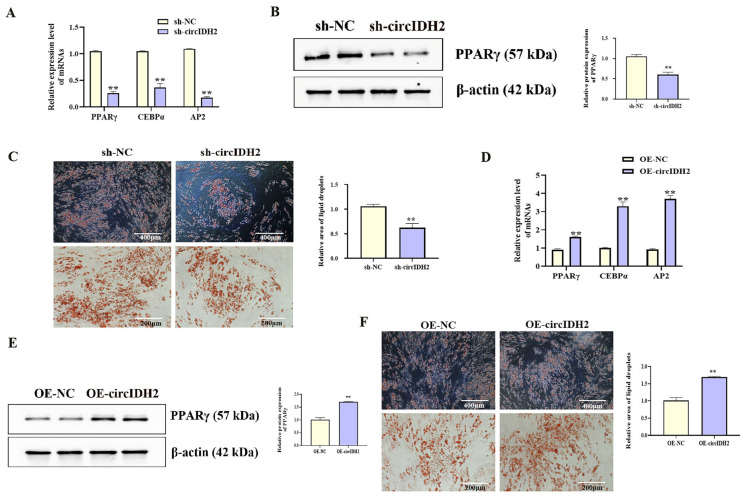
circIDH2-mediated regulation of adipogenic differentiation in porcine preadipocytes. (**A**) mRNA levels of adipogenic markers following interference (n = 3). (**B**) Protein levels of PPARγ following interference (n = 3). (**C**) Oil Red O staining after interference (n = 3). (**D**) mRNA levels of adipogenic markers following overexpression (n = 3). (**E**) Protein level of PPARγ following overexpression (n = 3). (**F**) Oil Red O staining after overexpression (n = 3). Abbreviations: sh-circIDH2, short hairpin RNA targeting circIDH2; sh-NC, negative control of short hairpin RNA; OE-circIDH2, overexpressing circIDH2; OE-NC, overexpressing negative control. ** *p* < 0.01.

With increasing differentiation days of porcine preadipocyte, the expression level of circIDH2 showed an upward trend (Figure 1A, *p* < 0.05). In addition, the effects of circIDH2 overexpression or interference on proliferation and adipogenic differentiation were assessed in porcine preadipocytes. Interference of circIDH2 significantly decreased its expression (Figure 1B, *p* < 0.01), confirming the efficiency of interference for subsequent experiments. Functional analyses revealed that circIDH2 interference significantly upregulated proliferation-related genes (Figure 1C, *p* < 0.01), increased viable cell numbers (Figure 1E, *p* < 0.01), and increased the proportion of EdU-positive cells (Figure 1D, *p* < 0.01). Moreover, circIDH2 interference suppressed both mRNA and protein levels of key adipogenic markers (Figure 2A,B, *p* < 0.01) and decreased lipid droplet accumulation (Figure 2C, *p* < 0.01). In contrast, circIDH2 overexpression produced the opposite effects (Figure 1F–I and Figure 2D–F). These findings demonstrated that circIDH2 suppressed proliferation while promoting adipogenic differentiation in porcine preadipocytes.

### 3.2. CircIDH2 Directly Sponges miR-193a-5p

**Figure 3:**
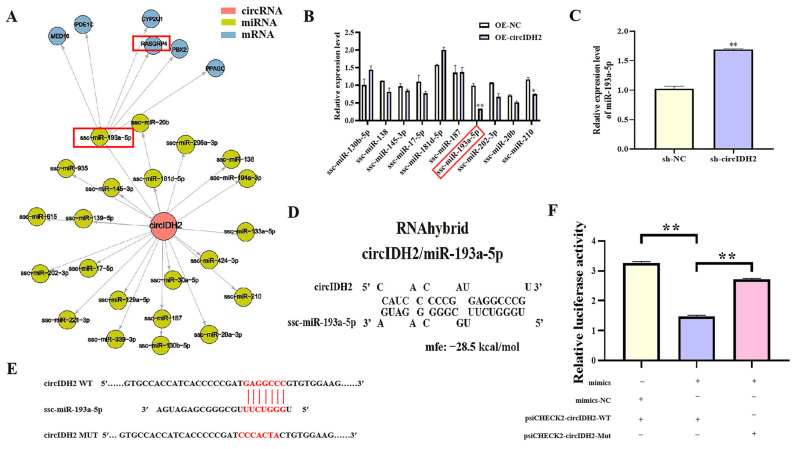
Interaction between circIDH2 and miR-193a-5p. (**A**) Regulatory network of circIDH2 and predicted miRNAs. (**B**) Changes in miRNAs expression following circIDH2 overexpression (n = 3). (**C**) miR-193a-5p expression after circIDH2 interference (n = 3). (**D**) Predicted complementary binding sites between circIDH2 and miR-193a-5p. (**E**) Wild-type and mutant vector sequences. (**F**) Dual-luciferase reporter assay validating the interaction between circIDH2 and miR-193a-5p (n = 3). Abbreviations: OE-circIDH2, overexpressing circIDH2; OE-NC, overexpressing negative control; sh-circIDH2, short hairpin RNA targeting circIDH2; sh-NC, negative control of short hairpin RNA; mimics, miR-193a-5p mimics; mimics-NC, negative control of mimics. (**A**,**B**) miRNA and mRNA boxed in red are the key targets of interest. (**E**) Bases highlighted in red indicate: the seed sequence of miR-193a-5p, the binding site within the circIDH2 sequence complementary to this seed sequence, and the mutated bases in the circIDH2 mutant sequence. * *p* < 0.05 and ** *p* < 0.01.

Bioinformatics analysis using TargetScan predicted potential miRNAs interacting with circIDH2, leading to the construction of a circIDH2-miRNA-mRNA regulatory network (Figure 3A). Among the candidate miRNAs, miR-193a-5p showed the most significant downregulation following circIDH2 overexpression (Figure 3B, *p* < 0.01), making it the focus of further mechanistic investigation. Validation experiments confirmed that circIDH2 interference significantly increased miR-193a-5p expression (Figure 3C, *p* < 0.01). RNAhybrid analysis revealed complementary base pairing between circIDH2 and the seed sequence of miR-193a-5p (Figure 3D). Dual-luciferase reporter assays further demonstrated this direct interaction: co-transfection of miR-193a-5p mimics with wild-type circIDH2 reporter plasmids significantly reduced luciferase activity, while mutant plasmids abolished this effect (Figure 3E,F, *p* < 0.01). Collectively, these results establish circIDH2 as a direct sponge for miR-193a-5p.

### 3.3. MiR-193a-5p Promotes Proliferation and Suppresses Adipogenesis in Porcine Preadipocytes

**Figure 4:**
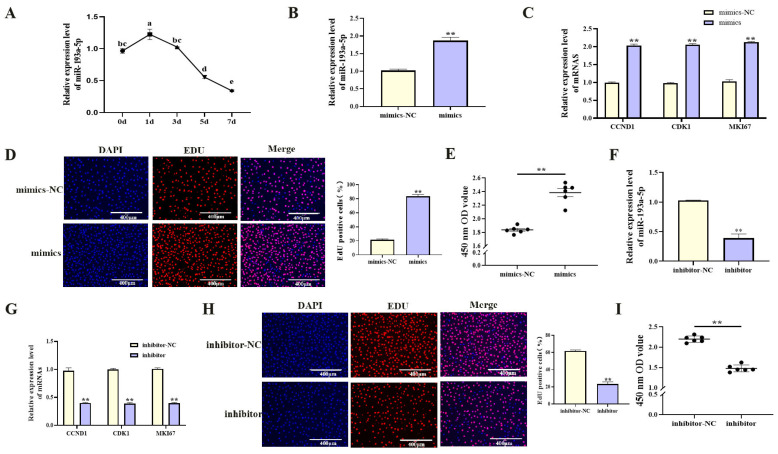
miR-193a-5p-mediated promotion of proliferation in porcine preadipocytes. (**A**) The expression patterns of miR-193a-5p during adipogenic differentiation in porcine preadipocytes (n = 3). (**B**) Transfection efficiency of miR-193a-5p mimics (n = 3). (**C**) Changes in mRNA expression of proliferation-related genes following transfection with miR-193a-5p mimics (n = 3). (**D**) EdU assay results after transfection with miR-193a-5p mimics (n = 3). (**E**) CCK-8 assay results after transfection with miR-193a-5p mimics (n = 6). (**F**) Transfection efficiency of miR-193a-5p inhibitor (n = 3). (**G**) Changes in mRNA expression of proliferation-related genes following transfection with miR-193a-5p inhibitor (n = 3). (**H**) EdU assay results after transfection with miR-193a-5p inhibitor (n = 3). (**I**) CCK-8 assay results after transfection with miR-193a-5p inhibitor (n = 6). Abbreviations: mimics, miR-193a-5p mimics; mimics-NC, negative control of mimics; inhibitor, miR-193a-5p inhibitor; inhibitor-NC, negative control of inhibitor. Different lowercase superscript letters indicate significant differences (*p* < 0.05). ** *p* < 0.01.

**Figure 5:**
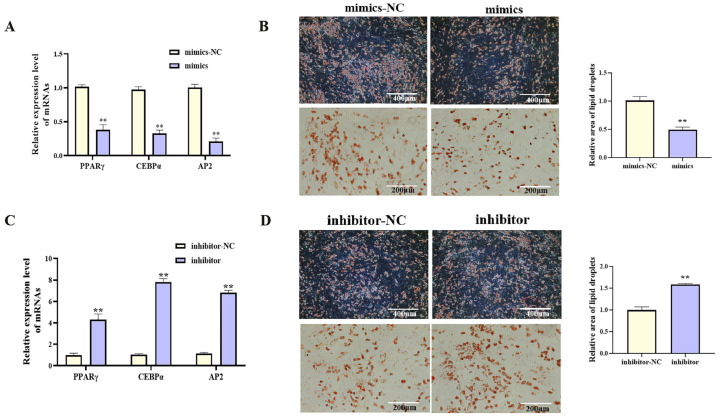
miR-193a-5p-mediated suppression of adipogenesis in porcine preadipocytes. (**A**) Changes in mRNA expression of adipogenic factors following transfection with miR-193a-5p mimics (n = 3). (**B**) Oil Red O staining showing lipid accumulation after transfection with miR-193a-5p mimics (n = 3). (**C**) Changes in mRNA expression of adipogenic factors following transfection with miR-193a-5p inhibitor (n = 3). (**D**) Oil Red O staining showing lipid accumulation after transfection with miR-193a-5p inhibitor (n = 3). Abbreviations: mimics, miR-193a-5p mimics; mimics-NC, negative control of mimics; inhibitor, miR-193a-5p inhibitor; inhibitor-NC, negative control of inhibitor. ** *p* < 0.01.

With increasing differentiation days of porcine preadipocyte, the expression level of miR-193a-5p showed an downward trend (Figure 4A, *p* < 0.05). Functional studies were performed by transfecting porcine preadipocytes with miR-193a-5p mimics or inhibitors. Transfection with miR-193a-5p mimics significantly increased miR-193a-5p expression (Figure 4B, *p* < 0.01), upregulated proliferation-related gene mRNA levels (Figure 4C, *p* < 0.01), increased viable cell counts (Figure 4E, *p* < 0.01), and increased the proportion of EdU-positive cells (Figure 4D, *p* < 0.01). During adipogenic differentiation, treatment with miR-193a-5p mimics markedly reduced the mRNA level of key adipogenic markers (Figure 5A, *p* < 0.01) and significantly reduced lipid droplet accumulation (Figure 5B, *p* < 0.01). In contrast, transfection with the miR-193a-5p inhibitor produced opposite effects (Figure 4F–I and Figure 5C,D). These results indicated that miR-193a-5p promoted proliferation and inhibited adipogenesis in porcine preadipocytes, acting antagonistically to circIDH2.

### 3.4. CircIDH2 Attenuates miR-193a-5p-Mediated Regulation of Proliferation and Adipogenesis in Porcine Preadipocytes

**Figure 6:**
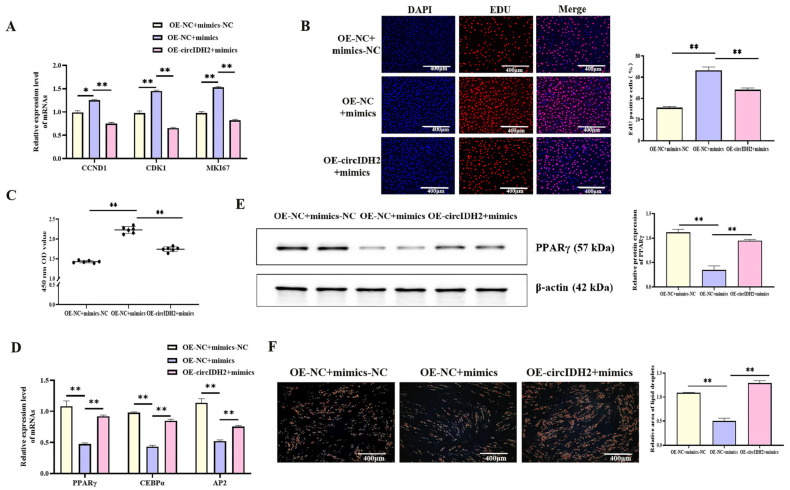
circIDH2 attenuation of miR-193a-5p-mediated regulation of proliferation and adipogenesis in porcine preadipocytes. (**A**) mRNA expression of proliferation-related genes (n = 3). (**B**) EdU staining results (n = 3). (**C**) CCK-8 assay results (n = 6). (**D**) mRNA expression of adipogenic markers (n = 3). (**E**) Protein levels of adipogenic factors (n = 3). (**F**) Oil Red O staining results (n = 3). Abbreviations: mimics, miR-193a-5p mimics; mimics-NC, negative control of mimics; OE-circIDH2, overexpressing circIDH2; OE-NC, overexpressing negative control. ** *p* < 0.01.

To investigate the functional interaction between circIDH2 and miR-193a-5p, porcine preadipocytes were co-transfected with OE-circIDH2 and miR-193a-5p mimics. qRT-PCR analysis showed that mRNA levels of proliferation-related genes were significantly downregulated in the co-transfection group compared with cells transfected with miR-193a-5p mimics alone (Figure 6A, *p* < 0.01). In addition, EdU staining and CCK-8 assays confirmed that the numbers of both viable cells and EdU-positive cells were markedly reduced in the co-transfection group relative to the mimics-only group (Figure 6B,C, *p* < 0.01), indicating that circIDH2 overexpression counteracted the proliferative effect induced by miR-193a-5p. In adipogenesis assays, both mRNA and protein levels of key adipogenic markers were significantly increased in the co-transfection group compared with the miR-193a-5p mimics-only group (Figure 6D,E, *p* < 0.01). Furthermore, Oil Red O staining showed a marked increase in lipid droplet accumulation in the co-transfection group (Figure 6F, *p* < 0.01). These findings indicated that circIDH2 antagonized the effects of miR-193a-5p by suppressing its pro-proliferative activity and relieving its inhibitory influence on lipid deposition.

### 3.5. MiR-193a-5p Directly Targets RASGRP4

**Figure 7:**
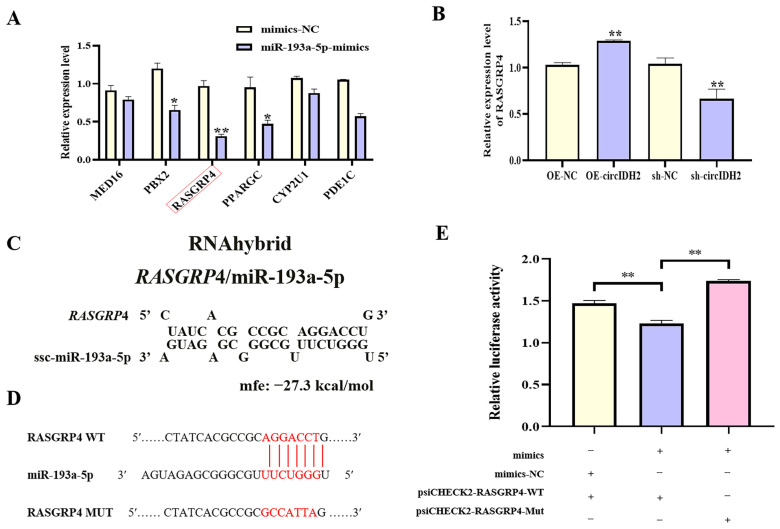
Validation of *RASGRP*4 as a direct target of miR-193a-5p. (**A**) Changes in expression of candidate target genes following miR-193a-5p overexpression (n = 3). (**B**) Effect of circIDH2 overexpression/interference on *RASGRP*4 mRNA levels (n = 3). (**C**) Predicted complementary binding sites between the *RASGRP*4 3′ UTR and miR-193a-5p. (**D**) Wild-type and mutant vector sequences. (**E**) Dual-luciferase reporter assay confirming direct targeting interaction (n = 3). Abbreviations: OE-circIDH2, overexpressing circIDH2; OE-NC, overexpressing negative control; sh-circIDH2, short hairpin RNA targeting circIDH2; sh-NC, negative control of short hairpin RNA; mimics, miR-193a-5p mimics; mimics-NC, negative control of mimics. (**A**) mRNA boxed in red are the key targets of interest. (**D**) Bases highlighted in red indicate: the seed sequence of miR-193a-5p, the binding site within the *RASGRP*4 sequence complementary to this seed sequence, and the mutated bases in the *RASGRP*4 mutant sequence. * *p* < 0.05 and ** *p* < 0.01.

To elucidate the downstream regulatory mechanism of miR-193a-5p, miRNA overexpression screening was combined with bioinformatics prediction using TargetScan, leading to the identification of six candidate target genes involved in lipid metabolism. Among these genes, *RASGRP*4 expression was significantly downregulated in cells transfected with miR-193a-5p mimics (Figure 7A, *p* < 0.01). Further experiments showed that circIDH2 overexpression markedly increased *RASGRP*4 mRNA levels (Figure 7B, *p* < 0.01), whereas circIDH2 interference significantly decreased *RASGRP*4 mRNA expression (Figure 7B, *p* < 0.01). RNAhybrid analysis identified complementary binding sites between the 3′ UTR of *RASGRP*4 and the seed sequence of miR-193a-5p (Figure 7C). Dual-luciferase reporter assays validated this interaction: co-transfection of miR-193a-5p mimics with the wild-type RASGRP4 3′ UTR reporter (WT) significantly decreased luciferase activity compared with the control (WT + mimics-NC) (Figure 7D,E, *p* < 0.01). This suppression was abolished when the binding sites were mutated (MUT + mimics) (Figure 7E). These results confirmed that miR-193a-5p directly targeted *RASGRP*4, revealing the circIDH2/miR-193a-5p/RASGRP4 axis as a potential regulator of lipid metabolism.

### 3.6. RASGRP4 Suppresses Proliferation and Enhances Adipogenic Differentiation in Porcine Preadipocytes

**Figure 8:**
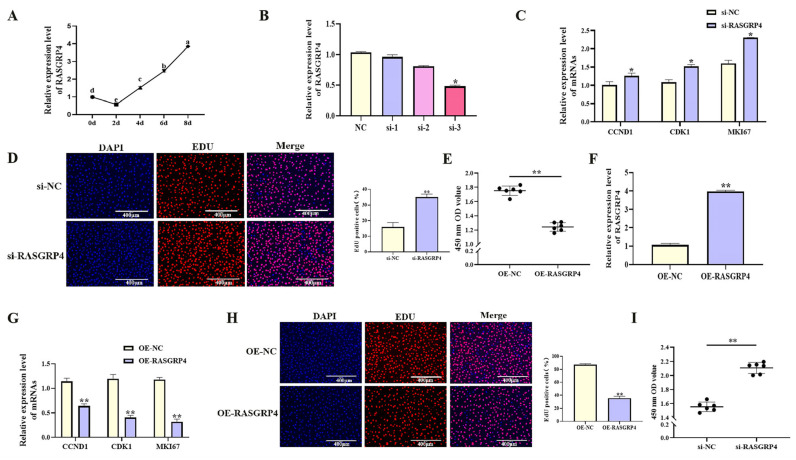
Regulatory roles of *RASGRP*4 in porcine preadipocyte proliferation. (**A**) The expression patterns of *RASGRP*4 during adipogenic differentiation in porcine preadipocytes (n = 3). (**B**) Efficiency of *RASGRP*4 interference (n = 3). (**C**) Expression of proliferation-related genes following *RASGRP*4 interference (n = 3). (**D**) EdU assay results after interference (n = 3). (**E**) CCK-8 assay results after interference (n = 6). (**F**) Efficiency of *RASGRP*4 overexpression (n = 3). (**G**) Expression of proliferation-related genes after overexpression (n = 3). (**H**) EdU assay results following overexpression (n = 3). (**I**) CCK-8 assay results following overexpression (n = 6). Abbreviations: si-1, si-2, and si-3 represent three distinct siRNA sequences targeting *RASGRP*4; si-*RASGRP*4, small interfering RNA targeting *RASGRP*4; si-NC, negative control of small interfering RNA; OE-*RASGRP*4, *RASGRP*4 overexpression construct; OE-NC, overexpressing negative control. Different lowercase superscript letters indicate significant differences (*p* < 0.05). * *p* < 0.05 and ** *p* < 0.01.

**Figure 9:**
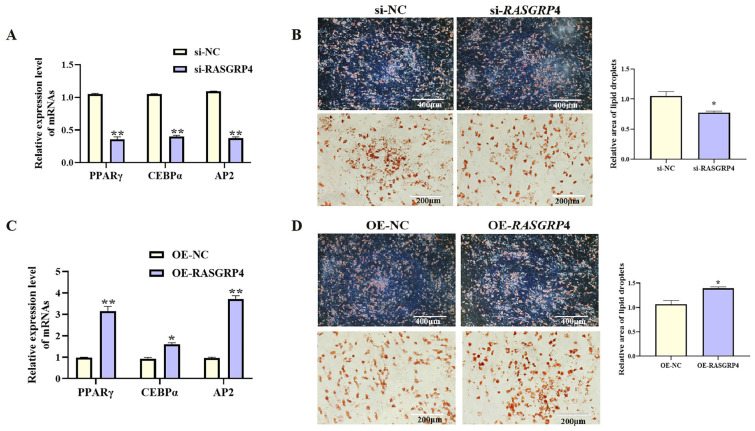
Regulatory roles of *RASGRP*4 in porcine preadipocyte differentiation. (**A**) mRNA levels of adipogenic factors following interference (n = 3). (**B**) Oil Red O staining following interference (n = 3). (**C**) mRNA levels of adipogenic factors after overexpression (n = 3). (**D**) Oil Red O staining results following overexpression (n = 3). Abbreviations: si-*RASGRP*4, small interfering RNA targeting *RASGRP*4; si-NC, negative control of small interfering RNA; OE-*RASGRP*4, *RASGRP*4 overexpression construct; OE-NC, overexpressing negative control. * *p* < 0.05 and ** *p* < 0.01.

With increasing differentiation days of porcine preadipocyte, the expression level of *RASGRP*4 showed an upward trend (Figure 8A, *p* < 0.05). The effects of *RASGRP*4 overexpression or interference on proliferation and adipogenic differentiation were evaluated in porcine preadipocytes. siRNA-mediated interference of *RASGRP*4 (si-3 group) significantly reduced its expression (Figure 8B, *p* < 0.05), and this group was selected for subsequent analyses. Functional assays showed that *RASGRP*4 interference markedly upregulated the expression of proliferation-related genes (Figure 8C, *p* < 0.05). Increased proliferative activity was further confirmed by CCK-8 assays (Figure 8E, *p* < 0.01) and EdU staining (Figure 8D, *p* < 0.01). Notably, *RASGRP*4 interference led to significant downregulation of key adipogenic markers at mRNA (Figure 9A, *p* < 0.01), accompanied by decreased lipid accumulation as shown by Oil Red O staining (Figure 9B, *p* < 0.05). In contrast, *RASGRP*4 overexpression produced the opposite effects (Figure 8F–I and Figure 9C,D). Collectively, these findings indicated that *RASGRP*4 inhibited proliferation and promoted adipogenic differentiation in porcine preadipocytes.

### 3.7. MiR-193a-5p Attenuates RASGRP4-Mediated Suppression of Proliferation and Promotion of Adipogenic Differentiation in Porcine Preadipocytes

**Figure 10:**
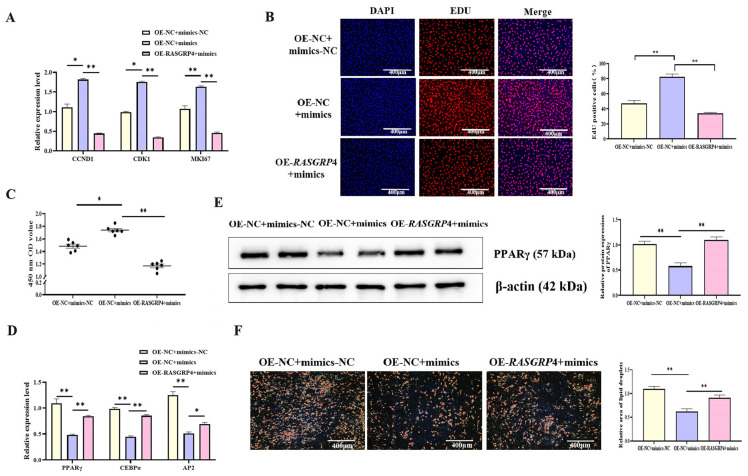
miR-193a-5p counteraction of *RASGRP*4-mediated regulation of proliferation and adipogenic differentiation in porcine preadipocytes. (**A**) Proliferation-related gene expression (n = 3). (**B**) EdU staining results (n = 3). (**C**) CCK-8 assay results (n = 6). (**D**) mRNA levels of adipogenic markers (n = 3). (**E**) Protein levels of adipogenic markers (n = 3). (**F**) Oil Red O staining results (n = 3). Abbreviations: mimics, miR-193a-5p mimics; mimics-NC, Negative control of mimics; OE-*RASGRP*4, *RASGRP*4 overexpression construct; OE-NC, overexpressing negative control. * *p* < 0.05 and ** *p* < 0.01.

To further investigate the regulatory interplay between miR-193a-5p and *RASGRP*4, rescue experiments were conducted. Transfection of miR-193a-5p mimics into porcine preadipocytes significantly promoted cell proliferation, as indicated by increased expression of proliferation-related genes (Figure 10A, *p* < 0.05), enhanced cell viability in the CCK-8 assay (Figure 10C, *p* < 0.05), and a higher proportion of EdU-positive cells (Figure 10B, *p* < 0.01). However, overexpression of *RASGRP*4 following miR-193a-5p mimic transfection markedly mitigated this pro-proliferative effect (Figure 10A–C, *p* < 0.01). Furthermore, miR-193a-5p mimics significantly suppressed adipogenic differentiation, as evidenced by decreased expression of adipogenic markers at both the mRNA (Figure 10D, *p* < 0.01) and protein levels (Figure 10E, *p* < 0.01), along with a reduction in lipid droplet formation (Figure 10F, *p* < 0.01). Notably, *RASGRP*4 overexpression effectively reversed the inhibitory effects of miR-193a-5p on adipogenesis (Figure 10D–F, *p* < 0.05).

## 4. Discussion

Adipose deposition in pigs plays a central role in determining key economic traits and influences the overall efficiency of swine production by affecting phenotypic characteristics such as carcass composition, meat quality, reproductive performance, and disease resistance [11,12,13,14]. As a vital metabolic and endocrine organ, adipose tissue is crucial for regulating meat quality and optimizing energy metabolism in livestock production [15]. Beyond its primary role in energy storage, adipose tissue functions as a multifunctional metabolic regulator, modulating systemic metabolism through the secretion of signaling molecules such as leptin and adiponectin [16]. Factors affecting adipose tissue deposition include nutritional, environmental, and genetic factors, among others. Extensive studies have demonstrated that circRNAs are important regulators of lipid metabolism [17,18]. This study focuses on the role of circRNAs in lipid metabolism and identifies a specific circRNA, circIDH2, which inhibits both the proliferation and adipogenic differentiation of porcine preadipocytes. These findings provide a theoretical basis for screening molecular targets to regulate adipose deposition in pigs.

The advent of high-throughput sequencing technologies has driven extensive research into circRNAs, revealing their crucial roles in various biological processes, including growth regulation [19,20], cellular differentiation [21,22,23], cancer diagnosis [24,25], and disease pathogenesis [26]. While the full spectrum of circRNA functions remains to be elucidated, growing evidence highlights their diverse regulatory mechanisms: acting as miRNA sponges, modulating transcriptional and translational programs, and interacting with RNA-binding proteins. Serving as central components of competing endogenous RNA (ceRNA) networks, circRNAs precisely regulate gene expression through their covalently closed structures—a distinctive feature of back-splicing that endows them with remarkable stability and tissue specificity [1]. By acting as molecular sponges that sequester miRNAs, circRNAs relieve repression of miRNA-targeted mRNAs, establishing dynamic circRNA/miRNA/mRNA regulatory axes that orchestrate downstream signaling pathways [27,28,29]. For example, hsa_circ_0001314 inhibits breast cancer proliferation and metastasis by sponging hsa-miR-548aj-3p, thereby activating the MAPK pathway [28]. Similarly, mmu_circ_0001874 regulates lipid deposition and inflammatory responses via miR-24-3p-mediated modulation of the Igf2/PI3K-AKT-mTOR signaling axis and the Igf2bp2/Ucp1 cascade [30]. In melanoma, hsa_circ_0079593 exhibits oncogenic activity through interaction with miR-516b-5p [31]. The previous studies by our research group have demonstrated that circHOMER1 inhibits porcine adipogenesis via the miR- 23b/SIRT1 axis [32], revealing that circIGF1R regulates myoblast differentiation via miR-16 [33]. Collectively, these findings highlight that circRNAs influence complex biological processes through ceRNA-mediated mechanisms. This study further demonstrates that circIDH2 regulates the proliferation and adipogenic differentiation of porcine preadipocytes via the miR-193a-5p/RASGRP4 axis, confirming that circIDH2 modulates porcine adipose deposition through ceRNA mechanisms. Critically, our findings establish a coherent regulatory axis where circIDH2, by sequestering miR-193a-5p, relieves the suppression of RASGRP4, ultimately tipping the balance of preadipocyte fate decision. This intricate interplay provides a molecular ex-planation for how circIDH2 exerts its inhibitory effect on porcine adipogenesis and fat deposition.

MiR-193a-5p has been widely studied in various disease contexts. In hepatocellular carcinoma, circ_0001806 regulates miR-193a-5p, which promotes tumor invasion by targeting MMP16 [34]. In colorectal cancer, its interaction with circCSPP1 influences epithelial–mesenchymal transition through COL1A1 [35]. In chronic obstructive pulmonary disease, circOSBPL2 sponges miR-193a-5p, leading to increased expression of its downstream target BRD4 and worsening inflammatory responses [36]. Furthermore, in pancreatitis models, circHipk3 activates the NLRP3 inflammasome by binding to miR-193a-5p [37]. However, the involvement of miR-193a-5p in lipid metabolism remains poorly explored. The present study demonstrates that miR-193a-5p promotes the proliferation of porcine preadipocytes while suppressing their adipogenic differentiation, thereby broadening the functional understanding of miR-193a-5p. Notably, this work provides the first direct evidence linking miR-193a-5p to the fundamental processes governing adipocyte development and lipid storage, uncovering a previously unrecognized role for this miRNA in adipose biology.

RAS guanyl-releasing protein 4 (RASGRP4), a member of the RASGRP family, is a guanine nucleotide exchange factor that specifically activates Ras proteins, regulating key biological processes such as cell proliferation, differentiation, and signal transduction [38]. Dysregulated expression of RASGRP4 has been associated with hematological disorders and tumorigenesis, including a potential role in acute myeloid leukemia [39,40]. Although direct evidence linking RASGRP4 to lipid metabolism regulation remains limited, its structural C1/DAG-binding domain strongly implies potential involvement via lipid signaling molecules. Given that DAG is both a key lipid metabolic intermediate and a critical ligand for PKC and RASGRP proteins, including RASGRP1, known to regulate T-cell receptor signaling via DAG-dependent membrane translocation [40], it warrants further investigation into whether RASGRP4 similarly modulates lipid metabolism-related pathways, such as insulin signaling or lipid synthesis. In the present study, RASGRP4 inhibits the proliferation of porcine preadipocytes while promoting their adipogenic differentiation.

## 5. Conclusions

**Figure 11:**
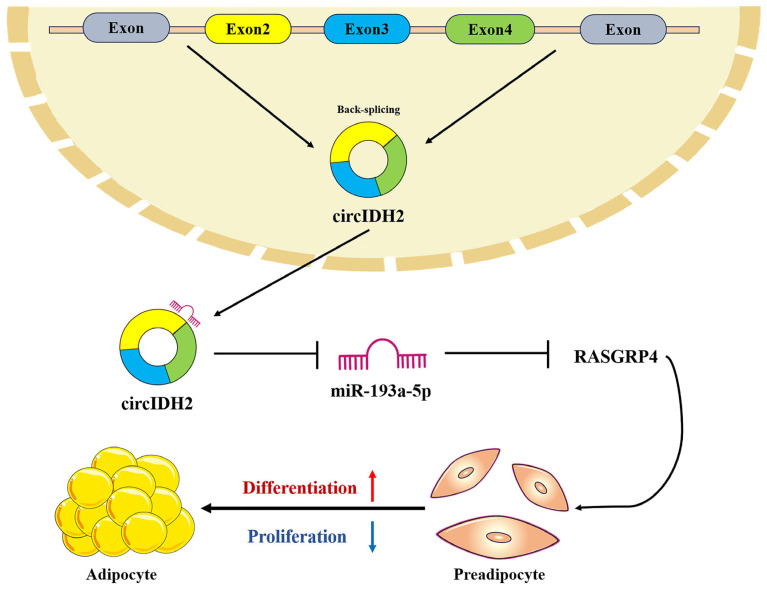
Mechanism diagram of circIDH2/miR-193a-5p/RASGRP4 regulation of proliferation and adipogenic differentiation in porcine preadipocytes.

In summary, this study identifies circIDH2 as a key regulator of porcine adipose deposition; it suppresses preadipocyte proliferation while promoting differentiation. Mechanistically, circIDH2 regulates adipose deposition through the miR-193a-5p/RASGRP4 axis (Figure 11). These findings offer valuable epigenetic insights into the genetic mechanisms underlying circIDH2-mediated adipose regulation in pigs, thereby enriching the understanding of the regulatory network governing porcine adipose development.

## Data Availability

The original contributions presented in this study are included in the article/Supplementary Materials. Further inquiries can be directed to the corresponding author.

## Supplementary Materials

The following supporting information can be downloaded at: https://www.mdpi.com/article/10.3390/cells14161265/s1, Table S1: Primer sequences of circRNA, mRNAs and miRNA.
