# Supplementary material for: CircIDH2 Modulates Porcine Adipogenesis via the miR-193a-5p/RASGRP4 Axis: Implications for ceRNA-Mediated Regulation of Fat Deposition
Source: Cells. 2025 Aug 15;14(16):1265. doi: 10.3390/cells14161265 (PMC12384935; doi:10.3390/cells14161265)
Supplement: Supplementary file 1 [file cells-14-01265-s001.zip › cells-3772198-supplementary.pdf]

Table S1 Primer sequences of circRNA, mRNAs and miRNA

Primers	species	Primer sequences (5'→3')
circIDH2	pig	F: GCATGGGCATGTACAACACAG<br>R: ATGGTGACCTGATCGTTGGTC
PCNA	pig	F: TGTAGCCGCGTCGTTGTGATTC<br>R: CGCTTCCAGCACCTTCTTCAGG
CDK1	pig	F: AACCACTTTTCCACGGGGATTCAG<br>R: GCTAGGCTTCCTGGTTTCCACTTG
MKI67	pig	F: AGCCCGTATCGTGTGCAAAA<br>R: CCTGCATCTGTGTAAGGGCA
CEBPα	pig	F: AGCCAAGAAGTCGGTAGA<br>R: CGGTCATTGTCACTGGTC
PPARγ	pig	F: AGAGTATGCCAAGAACATCC<br>R: AGGTCGCTGTCATCTAATTC
AP2	pig	F: AAGTCAAGAGCACCATAACC<br>R: GATACATTCCACCACCAACT
RASGRP4	pig	F: TGGCACAGCACCTGACTTAC<br>R: GAGGTGGAGTCGAAGGACTG
miR-193a-5p	pig	F: TGGGTCTTTGCGGGCGAGATGA<br>R: AACGCTTCACGAATTTGCGT
18S rRNA	pig	F: CCCACGGAATCGAGAAAGAG<br>R: TTGACGGAAGGGCACCA
U6	pig	F: CTCGCTTCGGCAGCACA<br>R: AACGCTTCACGAATTTGCGT
